# Preparation of endostatin-loaded chitosan nanoparticles and evaluation of the antitumor effect of such nanoparticles on the Lewis lung cancer model

**DOI:** 10.1080/10717544.2016.1247927

**Published:** 2017-02-06

**Authors:** Rui-Lin Ding, Fang Xie, Yue Hu, Shao-Zhi Fu, Jing-Bo Wu, Juan Fan, Wen-Feng He, Yu He, Ling-Lin Yang, Sheng Lin, Qing-Lian Wen

**Affiliations:** Department of Oncology, The Affiliated Hospital of Southwest Medical University, Luzhou, P.R. China

**Keywords:** Endostatin, nanoparticles, chitosan, lung cancer, drug carrier

## Abstract

The purpose of this study was to prepare ES-loaded chitosan nanoparticles (ES-NPs) and evaluate the antitumor effect of these particles on the Lewis lung cancer model. ES-NPs were prepared by a simple ionic cross-linking method. The characterization of the ES-NPs, including size distribution, zeta potential, loading efficiency and encapsulation efficiency (EE), was performed. An *in vitro* release test was also used to determine the release behavior of the ES-NPs. Cell viability and cell migration were assayed to detect the *in vitro* antiangiogenic effect of ES-NPs. In order to clarify the antitumor effect of ES-NPs *in vivo*, the Lewis lung cancer model was used. ES-NPs were successfully synthesized and shown to have a suitable size distribution and high EE. The nanoparticles were spherical and homogeneous in shape and exhibited an ideal releasing profile *in vitro*. Moreover, ES-NPs significantly inhibited the proliferation and migration of human umbilical vascular endothelial cells (HUVECs). The *in vivo* antiangiogenic activity was evaluated by ELISA and immunohistochemistry analyses, which revealed that ES-NPs had a stronger antiangiogenic effect for reinforced anticancer activity. Indeed, even the treatment cycle in which ES-NPs were injected every seven days, showed stronger antitumor effect than the free ES injected for 14 consecutive days. Our study confirmed that the CS nanoparticle is a feasible carrier for endostatin to be used in the treatment of lung cancer.

## Introduction

Tumor angiogenesis plays an important role in tumor growth and metastasis. Thus, targeting angiogenesis has become a promising option for the treatment of malignant diseases (Weis & Cheresh, 2011). Since the first report in 1997 (O'Reilly et al., 1997), endostatin (ES) has been known to specifically inhibit endothelial cell proliferation, migration and vessel formation (Zhuo et al., 2010), thereby inhibiting tumor growth. The systemic administration of ES was found to significantly inhibit the growth of animal tumors and human tumors in various mouse xenograft models (Ma et al., 2012, Dong et al., 2013). In 2005, the State FDA in China approved ES (Endostar) for the treatment of non-small-cell lung cancer. However, like many proteins, the biological half-life of ES is short due to its rapid metabolism, thus the plasma concentration of ES may fluctuate despite consecutive daily injection at a high dose during the first two weeks of a clinical treatment cycle (Kisker et al., 2001; Xu et al., 2007; Hu & Zhang, 2010; Chen & Hu, 2011). Short half-life and poor stability limit the clinical application of ES. Prolonged delivery of antiangiogenic therapy has been suggested as the most effective way to obtain long-term suppression of tumor angiogenesis and avoid tumor recurrence (Folkman et al., 2001; Kuroiwa et al., 2003). Thus, an effective drug delivery carrier with well-controlled release over an extended period of time may improve the tumor-killing activity of ES.

Nanoparticles (NPs) have attracted considerable attention as drug carriers due to their ability of overcoming the physiological barriers, as well as protecting and targeting the loaded substances to specific cells (Singh & Lillard, 2009). Naturally occurring polymers like chitosan (CS) can form NPs capable of carrying proteins and peptides (Katas et al., 2013; Kim et al., 2013). CS is a biodegradable polysaccharide that is derived from the deacetylation of chitin through a simple and mild preparation procedure. Its properties of low toxicity, good stability, controlled drug release and ability to overcome biological barriers have made CS NPs popular for drug delivery applications (Chen et al., 2015). It has been reported that CS NPs can protect the protein within the particle core (Amidi et al., 2010), avoiding degradation by enzymes and slowing filtration by the kidneys, thus increasing the protein retention in circulation. In previous studies, synthetic polymers such as PEG-PLGA (Hu & Zhang, 2010) and PLA (Du et al., 2015) were used to form long-acting NPs encapsulating ES. However, the preparation of these NPs required organic solvents to form w/o emulsions, which may adversely affect the stability of proteins. Unlike such polymers, the processes used to form CS NPs are simple and mild to proteins, as they do not involve the use of organic solvents and do not require high temperatures. CS also has an antitumor role by interfering with cell metabolism, inhibiting cell growth or inducing cell apoptosis (Wang et al., 2011; Karagozlu & Kim, 2014). Accordingly, using CS as the carrier to form ES-loaded NPs may improve its antitumor effects.

The objective of this study was to develop a long-term delivery system for ES using CS NPs, termed ES-loaded chitosan nanoparticles (ES-NPs). In addition, the antitumor effect of the produced ES-NPs was investigated in the Lewis lung cancer models.

## Materials and methods

### Materials

ES (11.2 mg/mL, Mw = 20 kDa) was provided by Shandong Simcere Medgeen Bio-Pharmaceutical Co. Ltd. (Shandong, China). Low molecular weight CS (Mw = 70 kDa (Mw/Mn = 1.5), Deacetylation Degree ∼92%), sodium tripolyphosphate (TPP), phosphate buffered saline (PBS) and trehalose were purchased from Sigma-Aldrich Inc. (St. Louis, MO). All other chemicals, including sodium hydroxide, glacial acetic acid and paraformaldehyde were analytical grade and used as received.

### Cell culture

Lewis lung carcinoma (LLC) cells and human umbilical vascular endothelial cells (HUVECs) were cultured in DMEM medium (HyClone, Thermo Scientific, Waltham, MA) and supplemented with 10% fetal calf serum (HyClone, Thermo Scientific, Waltham, MA). The cells were maintained at 37 °C in an incubator with a 5% CO_2_ atmosphere.

### Animals

All female C57BL/6J mice (4–5 weeks old) were provided by the Laboratory Animal Center of the Chongqing municipality (Chongqing, China). Animals were given sterile food pellets and water ad libitum, and were kept in a SPF laminar air flow box. All animal care and experimental procedures were approved and performed according to "Institutional Animal Care and Use Guidelines". The animal protocol used was reviewed and approved by the Institutional Animal Care and Use Committee of the Southwest Medical University (Luzhou, China).

### Preparation of ES-NPs

NPs were prepared by a simple ionic cross-linking method as described previously (Rampino et al., 2013). Briefly, 40 mg of CS was dissolved in 1% (v/v) acetic acid (1 mg/mL, pH 5) and TPP (1 mg/mL) was prepared in deionized water. Next, 0, 250, 500 or 1000 μL of ES was added to the CS solution. Then, 8 mL of the TPP solution was added dropwise into the CS solution with magnetic stirring at room temperature (RT) for 2 h to form NPs. All samples were prepared in triplicate.

The NPs were collected by centrifugation (Allegra 64R Centrifuge, Beckman-Coulter, Brea, CA) at 13 000 rpm for 30 min at 4 °C. The obtained sediment was resuspended in deionized water, and lyophilized by freeze drying (−55 °C, 48 h) using trehalose as the cryoprotectants. Subsequently, the dry ES-loaded NPs were weighed in an electronic weighing scale and sterilized by 25 kGy of ^60^Co gamma irradiation.

### Characterization of the ES-NPs

The size distribution and the zeta-potential of the produced ES-loaded NPs were measured with a NanoBrook 90Plus Zeta instrument (Brookhaven Instruments Corp., Holtsville, NY). The surface morphology of the NPs was investigated by transmission electron microscopy (TEM), using a Tecnai G2 F20 transmission electron microscope (FEI Company, Hillsboro, OR). Each sample was analyzed in triplicate.

### Drug-loading and encapsulation efficiency (EE) of ES-NPs

The supernatants obtained from the purification of the ES-NPs were collected to determine their loading and EE. The concentration of ES in the supernatant was measured using a Pierce Bicinchoninic acid Protein (BCA) assay (Beyotime Biotechnology, Shanghai, China). The results of the assay were validated using purified ES with a detection limit of 0.5–280 μg/mL. The amount of ES was calculated according to the standard curve (*R*^2^=0.9993). The loading efficiency (LE) and EE of the ES-NPs were expressed as follows:

$$\mathrm{LE}\%=\frac{\left(\mathrm{Total}\;\mathrm{amount}\;\mathrm{of}\;\mathrm{ES}\;\mathrm{added})-(\mathrm{Free}\;\mathrm{amount}\;\mathrm{of}\;\mathrm{ES}\right)}{\mathrm{ES}-\mathrm{loaded}\;\mathrm{NPs}\;\mathrm{dry}\;\mathrm{weight}}\times 100\%$$

$$\mathrm{EE}\%=\frac{\left(\mathrm{Total}\;\mathrm{amount}\;\mathrm{of}\;\mathrm{ES}\;\mathrm{added})-(\mathrm{free}\;\mathrm{amount}\;\mathrm{of}\;\mathrm{ES}\right)}{\mathrm{Total}\;\mathrm{amount}\;\mathrm{of}\;\mathrm{ES}\;\mathrm{added}}\times 100\%$$

### *In vitro* drug release and stability study of the ES-NPs

The *in vitro* release of ES from the ES-NPs was analyzed in PBS (pH = 7.4). Briefly, exact amounts of ES-NPs were dispersed in 2 mL of PBS and placed into test tubes at 37 °C with magnetic stirring. At the appropriate intervals, samples were centrifuged at 13 000 rpm for 30 min at 4 °C, the supernatants were collected and the pellet was resuspended in 2 mL of fresh medium. The amount of ES released from the CS-NPs was evaluated by means of the BCA assay, and the curve of ES release from the ES-NPs was then plotted.

In addition, to detect the stability of the carriers, the change in nanoparticle diameter in serum was examined over 48 h. The formulation stability of ES-NPs in 10% mouse serum containing PBS, incubated at 4 °C, 37 °C or RT, was evaluated by dynamic light scattering (DLS, NanoBrook 90Plus Zeta instrument, Brookhaven Instruments Corp., Holtsville, NY) after ultrasonic processing. Size changes were measured at pre-determined time points. Formulations were judged stable if no changes in particle size and no visual destabilization were observed, such as creaming, phase separation or presence of compact aggregates.

### Cell viability assay

HUVECs were seeded into 96-well plates at 1 × 10^4^ cells/well for 12 h, and then incubated with ES (200 μg/mL), ES-NPs (contained 200 μg/mL ES) or blank CS NPs for 24, 48 and 72 h. HUVECs without treatment served as a control. Cell viability was determined by the MTT assay and calculated as the % of cells relative to the number of cells in the control.

### Transwell migration assay

Cell migration was evaluated based on the ability of the cells to migrate across a transwell filter (8-μm pores, Costar, Cambridge, MA). A total of 4 × 10^4^ HUVECs suspended in serum-free DMEM were added to the upper chamber, and DMEM medium containing 10% fetal bovine serum was added to the lower chamber. Next, ES (200 μg/mL), ES-NPs (containing 200 μg/mL ES), blank CS NPs and PBS were added into the top chambers in separate experiments. After a 12 h or 24 h incubation at 37 °C, the non-migrated cells were scraped off of the filter using a cotton swab and the cells that migrated to the lower side of the upper chamber were fixed with 4% paraformaldehyde and stained with crystal violet. The cells per microscopic field (400×) were imaged and counted in 10 randomly chosen fields.

### Tumor inhibition effect by ES-NPs *in vivo*

The subcutaneous lung cancer model was established by injecting 100 μL suspension of LLC cells (1 × 10^7^ cells/mL) into the right armpit of C57BL/6J mice. The cells were allowed to grow for two weeks until the tumors were approximately 200 mm^3^ in volume. Then, the tumor-bearing mice were randomly assigned to six groups (*n* = 10 each): control, ES, ES-NPs1, ES-NPs2, ES-NPs3 and blank CS NPs. All drugs were administrated via i.p. injection according to the regimen shown in Figure 4(A). Based on clinical doses used in humans and the results from a previous study (Fan et al., 2015), ES 10 mg/kg/day was administered once daily for 14 consecutive days. For the three ES-NPs groups, ES-loaded nanoparticles containing the same amount of ES were injected every seven days (ES-NPs1), every two days (ES-NPs2) or every day (ES-NPs3). In the control and blank CS NPs groups, the same volume of PBS or blank CS NPs, respectively, were injected. Mice were sacrificed by cervical dislocation on day 21 and the tumor tissues and blood samples were collected for further analysis. During the treatment, tumor size was measured by calipers (length and width) every two days. The tumor volumes were calculated with the formula *V* = *a*×*b*^2^×π/6, where *a* is the larger and *b* is the perpendicular shorter tumor axis. A tumor growth curve was plotted based on tumor size and the length of survival, in days, after treatment. The tumor volume inhibition rate on day 21 was calculated according to the following equation:

$$\mathrm{Inhibition}\;\mathrm{rate}\%=\left(1-\frac{(\begin{array}{c} {\mathrm{Volume}}_{\mathrm{Day}\;1\;\mathrm{experiment}\;\mathrm{group}}-\\ {\mathrm{Volume}}_{\mathrm{Day}\;21\;\mathrm{experiment}\;\mathrm{group}} \end{array})}{(\begin{array}{c} {\mathrm{Volume}}_{\mathrm{Day}\;1\;\mathrm{control}\;\mathrm{group}}-\\ {\mathrm{Volume}}_{\mathrm{Day}\;21\;\mathrm{control}\;\mathrm{group}} \end{array})}\right)\times 100\%$$

### Enzyme-linked immunosorbent assay (ELISA) analysis

Blood samples of each group were collected in Eppendorf tubes. After immediate centrifugation (1800×*g*) for 10 min at 4 °C, plasma was separated and then frozen immediately at −80 °C until analysis. Plasma ES and vascular endothelial growth factor (VEGF) levels were measured by an ELISA kit according to manufacturer’s instructions (RayBiotech Inc., Norcross, GA). PBS solution was used as a control and absorbance was measured at 450 nm. The concentrations of ES and VEGF were calibrated with the ES and VEGF standard curve.

### Immunohistochemistry

Tumor tissue samples harvested from the sacrificed mice were fixed in 10% formalin, paraffin-embedded and sectioned. Tissue sections 5 μm thick were dewaxed and incubated with 0.01 M sodium citrate for antigen retrieval. The slides were rinsed in PBS and incubated overnight at 4 °C with rabbit antimouse CD31 primary antibodies (Bio-World, Dublin, OH). Biotinylated goat anti-rabbit anti-immunoglobulin G (Ig G) was used as the secondary antibody. Steps were then performed using the immunostaining kit following manufacturer’s instructions. Quantification of the microvessel density (MVD) was independently assessed according to the Weidner method (Weidner et al., 1991) by two observers. Briefly, the sections were screened at lower magnifications (100×) to identify three most vascularized areas (hot spots). Microvessels were counted in these areas at a magnification of 400×, and the average number of microvessels was recorded.

### Statistical analysis

The continuous variables were expressed as mean ± SD. The significance of the differences between groups were determined by one-way analysis of variance (ANOVA) and the average number of pairwise comparisons was determined by Tamhane's T2 test; *p* values of less than 0.05 were considered statistically significant. Data analyses were performed using the SPSS statistics 23.0 software (IBM Corp., Armonk, NY).

## Results

### Characteristics of ES-loaded NPs

**Figure 1.**
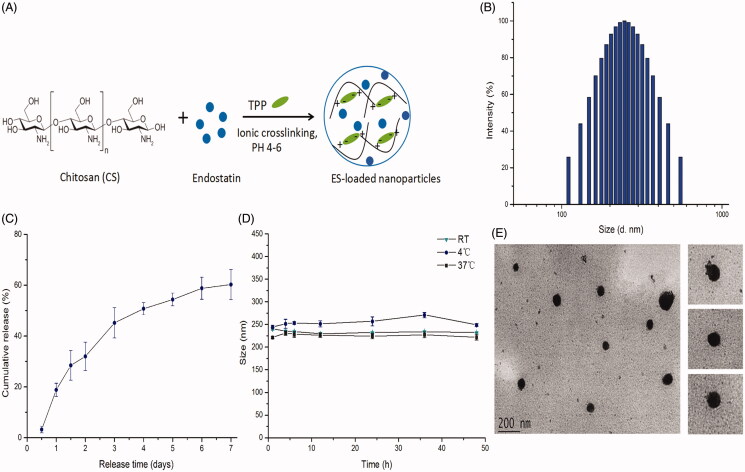
The characteristics of endostatin-loaded nanoparticles. (A) The fabrication process of ES-NPs. Endostatin-loaded chitosan nanoparticles were prepared by ionic cross-linking method with dropwise addition of TPP to a chitosan solution. (B) The size distribution of our chosen ES-NPs. The results showed that the particles were 246.89 ± 3.5 nm in diameter. (C) The release behavior of ES-NPs *in vitro*. The endostatin release profile was biphasic, with an initial abrupt release and a subsequent sustained release. (D) The formulation stability of ES-NPs in mouse serum at 4 °C, 37 °C or room temperature (RT). (E) TEM images of ES-NPs. Transmission electron microscopy showed that nanoparticles were round particles with relative smooth edges.

In this study, ES-NPs were prepared by an ionic cross-linking method with dropwise addition of TPP to a CS solution (Figure 1A). Four samples of ES-NPs containing different amount of ES were prepared, as shown in Table 1. The results revealed that the blank CS NPs (lacking any drug; sample 1) had a mean diameter of 211.50 ± 1.58 nm. After loading with ES, the diameter of the NPs was increased, in a manner that was dependent on the amount of ES added. Additionally, an upward trend in LE% was observed as the amount of ES was increased from 250 to 1000 μL. However, for each sample, higher LE% value corresponded to a lower EE% value. Based on these results, we selected sample 3 (500 μL of ES) for subsequent experiments. The drug LE of these ES-NPs was 10.70 ± 0.16% and the EE was 74.81 ± 4.23%. The morphological analysis of these ES-NPs showed a spherical structure with a relatively smooth surface (Figure 1E). The particles were 246.89 ± 3.5 nm in diameter (Figure 1B). The PDI was 0.285 ± 0.008 and the zeta potential was −36.34 ± 0.16 mV.

**Table 1.** Characteristics of four endostatin-loaded nanoparticles.

No.	Amount of ES (μL)	Size (nm)	PDI	Zeta potential (mV)	EE (%)	LE (%)
1	0	211.50 ± 1.58	0.223 ± 0.005	−38.36 ± 0.11	–	–
2	250	227.31 ± 2.64	0.238 ± 0.017	−38.17 ± 0.41	78.25 ± 2.10	6.08 ± 0.16
3	500	246.89 ± 3.50	0.285 ± 0.008	−36.34 ± 0.16	74.81 ± 4.23	10.74 ± 0.16
4	1000	247.91 ± 2.38	0.289 ± 0.001	−32.21 ± 0.32	62.42 ± 2.90	11.46 ± 0.54

### Release and stability of ES-loaded NPs *in vitro*

The release of ES from the ES-NPs in PBS (pH 7.4) is shown in Figure 1(C), where it is evident that the ES release profile is biphasic, with an initial abrupt release and a subsequent sustained release. A total of 28.42% ± 5.82% of the loaded ES was released during the initial 36 h. Almost 40% of the loaded ES remained enveloped in the NPs on day 7, and the drug release was 60.22% ± 5.95%.

To further examine the formulation stability of ES-NPs, the change in nanoparticle diameter in the presence of serum proteins was tested over 48 h at different temperature. As shown in Figure 1(D), there was no obvious change in the mean diameter in each group, indicating that the NPs were stable in mouse serum.

### The effects of ES-NPs on cell viability *in vitro*

**Figure 2.**
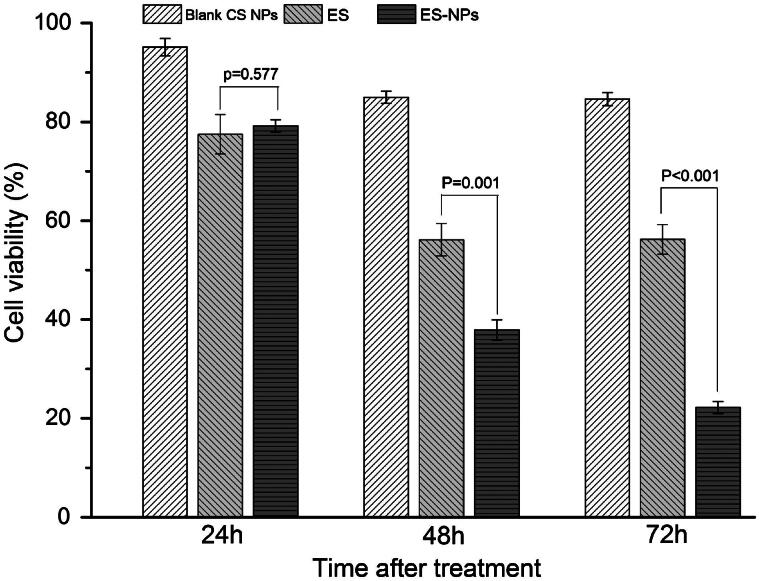
The effects of ES-NPs on cell viability *in vitro*. HUVECs were treated with ES (200 μg/mL), ES-NPs (contained 200 μg/mL ES) or blank CS NPs for 24 h, 48 h and 72 h. The data showed that ES-NPs had strong effect on inhibiting the proliferation of HUVECs.

The MTT assay was used to examine the effects of ES-NPs on HUVECs viability *in vitro* (Figure 2). After treatment with ES-NPs for 24 h, the cell viability was slightly reduced. In fact, the result was similar to the viability of cells treated with free ES (*p* = 0.577). However, with longer treatment times, the difference between the ES-NPs and ES groups became significantly larger (37.88% ± 2.06% versus 56.10% ± 3.26%, *p* = 0.001; 22.20% ± 1.22% versus 56.19% ± 3.00%, *p* < 0.001). The results confirmed our hypothesis that ES-NPs had a strong inhibitory effect on cell viability *in vitro*. On the other hand, blank CS NPs showed very low inhibition of HUVECs for any length of time tested, indicating that the inhibitory effect of the ES-NPs on cells resulted most likely from the release of ES from the NPs.

### The effects of ES-NPs on cell migration *in vitro*

**Figure 3.**
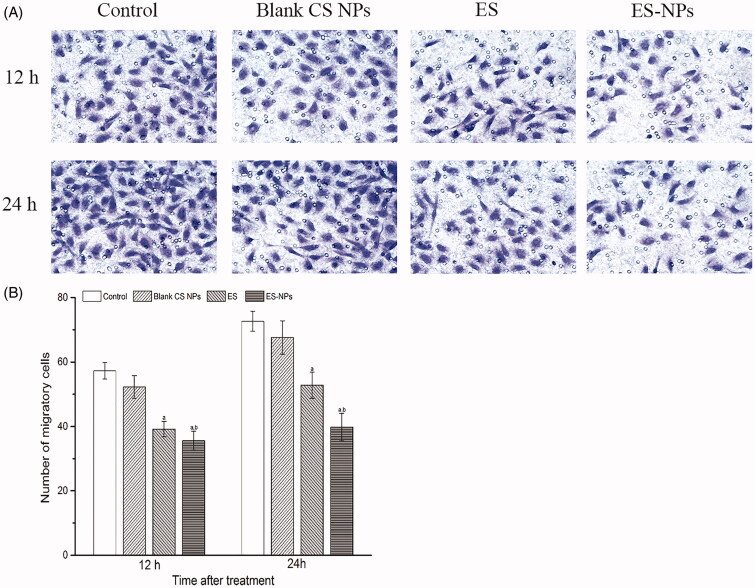
The effects of ES-NPs on cell migration *in vitro*. The cells were incubated with PBS, ES, ES-NPs or blank CS NPs for 12 h and 24 h. The data showed that ES-NPs had a significant effect on HUVECs migration. ^a^*p* < 0.05 versus control; ^b^*p* < 0.05 versus ES group.

We next evaluated the effects of ES-NPs on HUVECs migration using a transwell assay. After 12 h or 24 h treatment, the migration of HUVECs treated with ES-NPs was significantly inhibited compared to the control, blank CS NPs and free ES (Figure 3). The numbers of migratory cells were 39.2 ± 2.4 for ES and 35.6 ± 2.9 for ES-NPs (*p* = 0.039) at 12 h after treatment. Moreover, the difference between the two groups increased at 24 h (52.5 ± 4.0 versus 39.8 ± 4.2, *p* < 0.001). Although ES showed a significant effect on HUVECs migration, the effect of ES-NPs was stronger.

**Figure 4.**
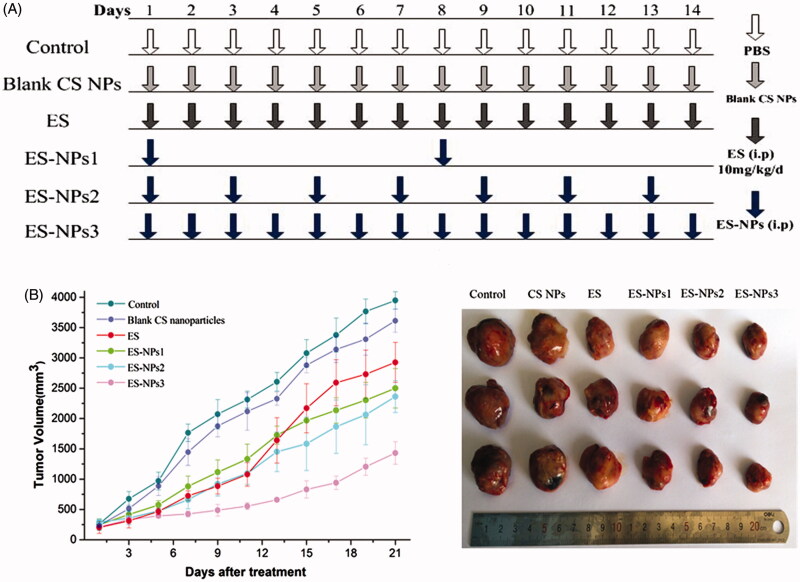
Tumor volume changes in each group. (A) Treatment schedule. Two weeks after inoculation, the tumor-bearing mice were randomly assigned to six groups: control, ES, ES-NPs1, ES-NPs2, ES-NPs3 and blank CS nanoparticles (*n* = 10). (B) Tumor growth curve in each group. (C) The final tumor volume on day 21.

### Tumor inhibition effect of ES-NPs *in vivo*

The tumor formation rate was 100% in this study. There were no significant differences in the body weight of mice between the six groups before and after treatment (data not shown). The tumor growth occurred rapidly in the control and blank CS NPs groups, but was significantly repressed in the ES and three ES-NPs groups as shown in Figure 4(B). In addition, we found that the tumor volume of the ES group was similar to that of ES-NPs1 or ES-NPs2 group during the initial 11 days after treatment. However, the tumors in the ES group started to grow rapidly on day 13, and the final tumor volume was significant larger than that in the ES-NPs1 or ES-NPs2. Additionally, the inhibition rate on day 21 was 22.67% in the ES group, 41.74% in the ES-NPs1 group, 43.76% in the ES-NPs2 group and 8.40% in the blank CS NPs group. Thus, the two ES-NPs groups showed better antitumor effect. In contrast, the tumor growth of ES-NPs3 was slow throughout the whole course of treatment, and the final inhibition rate was 66.91%. These results indicated that the treatment with ES-NPs increased the antitumor effect of ES, and even the effect of ES-NPs1 (ES-NPs injected every seven days) was stronger than that of free ES.

### The effects of ES-NPs in tumor angiogenesis

To further verify our findings, the microvascular density in each group was assessed by counting the number of CD31-positive cells. Representative immunohistochemistry stained samples of CD31 are shown in Figure 5(A). The amount of microvessels were high in the control and blank CS NPs groups, but were decreased in the ES and three ES-NPs groups (Figure 5B). The lowest MVD was observed in the ES-NPs3 group, and this value was significantly different relative to the control (*p* < 0.001) and free ES (*p* < 0.001).

**Figur 5.**
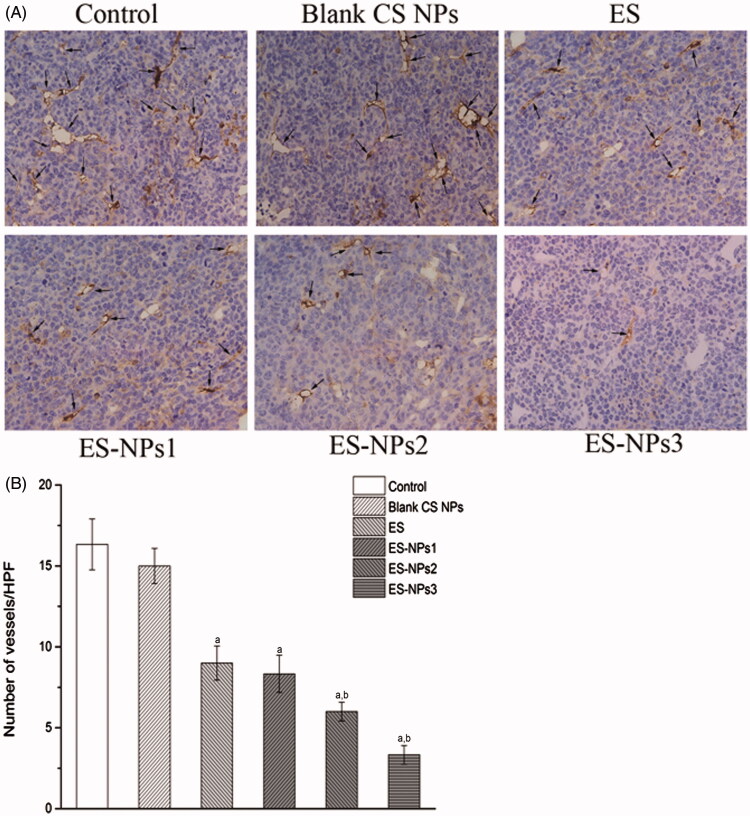
The microvascular density (MVD) in each group. (A) Tumor vessels were stained darkly by CD31 antibody as arrows indicated (×400). (B) Histogram of mean microvascular density in each group. ^a^*p* < 0.05 versus control; ^b^*p* < 0.05 versus ES group.

**Figure 6.**
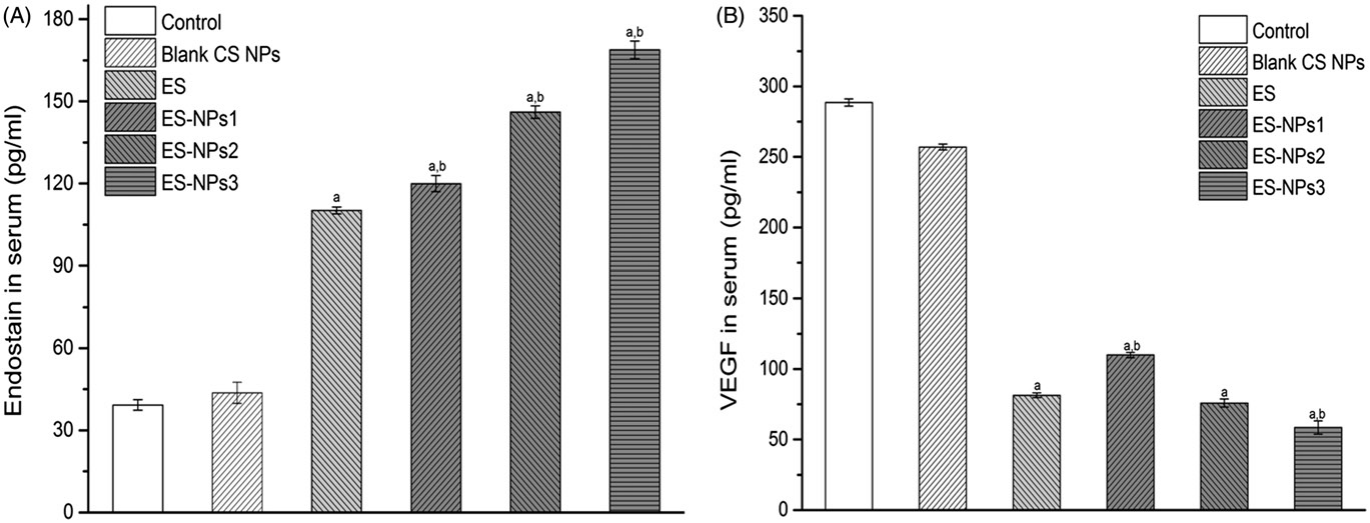
The serum endostatin (A) and VEGF (B) levels of each group. Mice in each group were sacrificed on day 21, and the blood samples were collected to detect the serum endostatin and VEGF levels by ELISA. ^a^*p* < 0.05 versus control; ^b^*p* < 0.05 versus ES group.

The levels of ES and VEGF in serum were also evaluated at the end of the treatment cycle (Figure 6). As expected, the serum level of ES in the ES-NPs3 group was much higher than that detected in the control (*p* < 0.001), blank CS NPs (*p* < 0.001) and ES groups (*p* < 0.001). Even the serum ES levels in the ES-NPs1 and ES-NPs2 were much higher than that detected in free ES. In addition, we also observed the lowest VEGF level in the ES-NPs3 group.

## Discussion and conclusions

Antiangiogenic therapy is an emerging research field and a new clinical strategy in tumor-targeting therapy (Stoll et al., 2003). ES has been shown to effectively suppress tumor growth by inhibiting angiogenesis. Similar to many protein or peptide drugs, ES can be easily degraded by enzymes *in vivo* and has poor permeability, stability and a short half-life (Han et al., 2011; Cui et al., 2013). Methods, such as the preparation of PEGylated ES (Tong et al., 2010), ES microsphere (Wu et al., 2009) and ES-loaded NPs have been used to try to overcome these shortcomings. Although ES-loaded PEG-PLGA (Hu & Zhang, 2010) and PLA (Du et al., 2015) NPs were reported previously, in this work, we have prepared new ES NPs, named ES-NPs, and evaluated their characteristics and antitumor effects.

The mean diameter of the ES-NPs chosen to conduct our experiments in this study was 246.89 ± 3.5 nm, indicating that they could be administered intravenously or intraperitoneally. The zeta potential of the chosen ES-NPs was −36.34 ± 0.16 mV, suggesting that the delivery system was stable (Ahmed & Aljaeid, 2016). As ES is a 20 kDa peptide, the encapsulation of ES into NPs is difficult, but we observed a high encapsulation rate in the ES-loaded CS NPs at 74.81%±4.23%. Our results confirmed that CS is suitable to encapsulate protein and peptide, consistent with previous studies (Katas et al., 2013; Piras et al., 2015).

An *in vitro* release test was used to determine the ES release profile from the prepared CS NPs. It is established that the mechanism of drug release from CS NPs is related to (a) the amount of drug present on the particle surface, (b) drug diffusion from the CS matrix and (c) CS degradation and erosion (Agnihotri et al., 2004; Ahmed & Aljaeid, 2016). We observed an abrupt release of ES-NPs due to the release of the drug from the surface layer of the particle. After this initial fast release, a sustained release occurred (Figure 1C). The final drug release was 60.22%±5.95% after seven days. Our data suggested that the ES-NPs have excellent controlled release function, and the drug release speed conformed to the therapeutic needs.

The role of ES on tumor angiogenesis has been confirmed in previous studies (Ling et al., 2007; Fu et al., 2011; Xu et al., 2014). To determine the anti-angiogenic activity of ES-NPs *in vitro*, the cell viability and cell migration assays were performed. Like in other studies (Xu et al., 2014), we observed a significant inhibitory effect of ES on HUVECs proliferation and migration *in vitro*, but the effects exerted by ES-NPs appeared to be stronger. Additionally, with the extension of the treatment time, the difference between the two groups increased (Figures 2 and 3). A possible explanation for such results is that temperature, pH or other factors have been thought to possibly influence the stability of ES (Nie et al., 2006). Moreover, the ES-NPs produced and used in our study may improve the stability and release of ES in a sustained manner. Accordingly, we observed that ES encapsulated into NPs exhibited a better antiangiogenic effect than free ES *in vitro*, which was consistent with work by Du et al. (2015). Furthermore, CS could promote the contact between the protein drug and biomembranes (Wang et al., 2011), thereby improving the availability of the protein drug, which may also contribute to the results we have obtained.

Antiangiogenic therapy is one of the major strategies for lung cancer treatment. In a phase III clinical trial of ES in China, the combination of ES and chemotherapy was demonstrated to significantly improve the overall and progression-free survival of advanced NSCLC (Wang et al., 2005). In the present study, we used a lung cancer model to examine whether the antitumor effect of ES was improved when incorporated into CS NPs. As anticipated, ES-NPs significantly improved tumor growth inhibition in our study. Even the ES-NPs injected every seven days showed stronger antitumor effect than free ES injected for 14 consecutive days. It is well documented that antiangiogenic therapy requires daily administration to achieve tumor inhibition (Kisker et al., 2001). Our data suggest that the use of CS NPs could reduce the amount and administration frequency of ES needed to achieve significant tumor inhibition in mice. Furthermore, the daily injection of ES-NPs achieved the best antitumor effect, with a tumor inhibition rate of 66.91%.

Since the major role of ES is to inhibit neovascularization or induce apoptosis of vascular endothelium, the MVD in each group was assessed separately. The data indicated that microvascular densities were decreased in the three ES-NPs groups (Figure 5), suggesting that the ES-NPs improved antitumor activity by inhibiting tumor angiogenesis *in vivo*. CS NPs could protect ES and prolong its retention in circulation. Accordingly, we observed that the serum ES levels in the ES-NPs1, ES-NPs2 or ES-NPs3 groups were much higher than that detected in the serum of the free-ES group (*p* < 0.05, Figure 6A). This may also explain our MVD results. It is widely known that the antiangiogenic effect of ES is related to the VEGF activity. ES has been shown to block the VEGF-induced tyrosine phosphorylation of KDR/Flk-1 (Ling et al., 2007), and also to down-regulate the expression of VEGF (Folkman, 2006). Consistent with such findings, a low serum VEGF level was found in ES group, while the serum VEGF level was lowest in the ES-NPs3 group (Figure 6B). Previous studies have found that CS or CS NPs could inhibit tumor growth *in vivo* (Maeda & Kimura, 2004), However, in this study, we did not observe any evident antitumor effect of blank CS NPs; actually, the tumor inhibition rate was only 8.40% on day 21.

It has been reported that the antitumor activity of ES is biphasic and operates in a U-shaped curve (Celik et al., 2005). Circulating levels of ES that are too high or too low are inactive (Kuo et al., 2001; Pawliuk et al., 2002). This U-shaped response might result from receptor desensitization (such as integrins). In this study, during a two-week treatment cycle, the ES-NPs were injected every seven days, every two days or every day. All three ES-NPs groups exhibited significant antitumor and antiangiogenic effects with no toxicity. This result suggested that levels of circulating ES, released from the NPs, were adequate in the three ES-NPs groups, neither too low nor too high. Although daily injection of ES-NPs resulted in the best antitumor effect in this study, the optimal administration plan and suitable treatment doses of ES-NPs must be determined in further studies.

In conclusion, in this study, ES-loaded CS NPs were successfully synthesized using the ionic cross-linking method. The NPs released ES in a sustained manner *in vitro* and showed an excellent inhibitory effect on HUVECs proliferation and migration. Although ES-NPs significantly improved the anticancer activity of ES by affecting angiogenesis, many other characteristics remain to be investigated.
